# Supplementary material for: Strain-engineering Mott-insulating La2CuO4
Source: Nat Commun. 2019 Feb 19;10:786. doi: 10.1038/s41467-019-08664-6 (PMC6381167; doi:10.1038/s41467-019-08664-6)
Supplement: Supplementary file 1 — Supplementary Information [file 41467_2019_8664_MOESM1_ESM.pdf]

Strain-Engineering Mott-Insulating La_2CuO_4

O. Ivashko *et al.*

I. SUPPLEMENTARY FIGURES

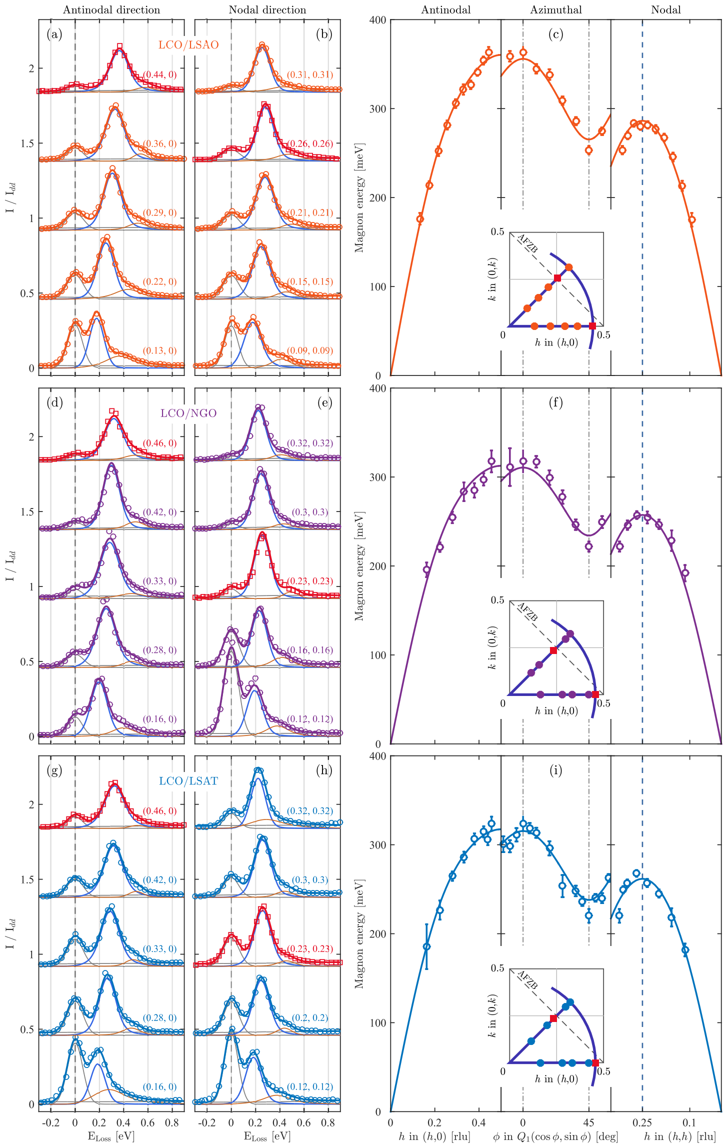

Supplementary Figure 1. **Magnon Dispersion of thin films.** In (a) and (b) are presented raw spectra of LCO/LSAO along with the fits (and its single components) for antinodal and nodal directions respectively. Similar data are presented for LCO/NGO in (d) and (e) and for LCO/LSAT in (g) and (h). Each spectra is at the **q** vector as indicated, which is also schematically illustrated in the respective insets. Magnon dispersions and the respective Hubbard model fits are presented for LCO/LSAO, LCO/NGO and LCO/LSAT in (c), (f) and (i) respectively, for direction as indicated. The error bars are three times the standard deviation obtained from the fits. In (c), (f) and (i) Q_1 takes different values for each compound due to different incident energies and in-plane lattice parameters, resulting in 0.4437 for LCO/LSAO, 0.4564 for LCO/NGO and 0.4568 for LCO/LSAT. Source data are provided as a Source Data file.

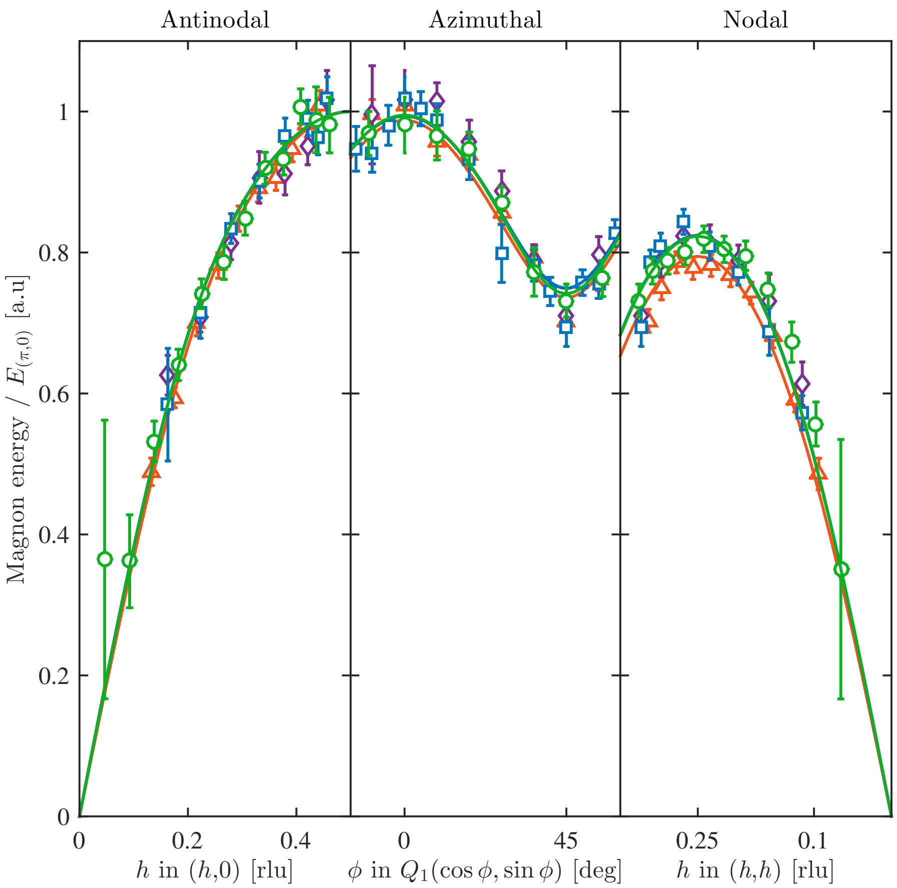

Supplementary Figure 2. **Scaling the Magnon Dispersions.** Dispersion of the magnetic excitations for all the measured samples (LCO/LSAO, LCO/NGO, LCO/LSAT and LCO/STO) scaled to the maximum along the antinodal direction $E_{(\pi,0)}$. The error bars are three times the standard deviation obtained from the fits. Source data are provided as a Source Data file.

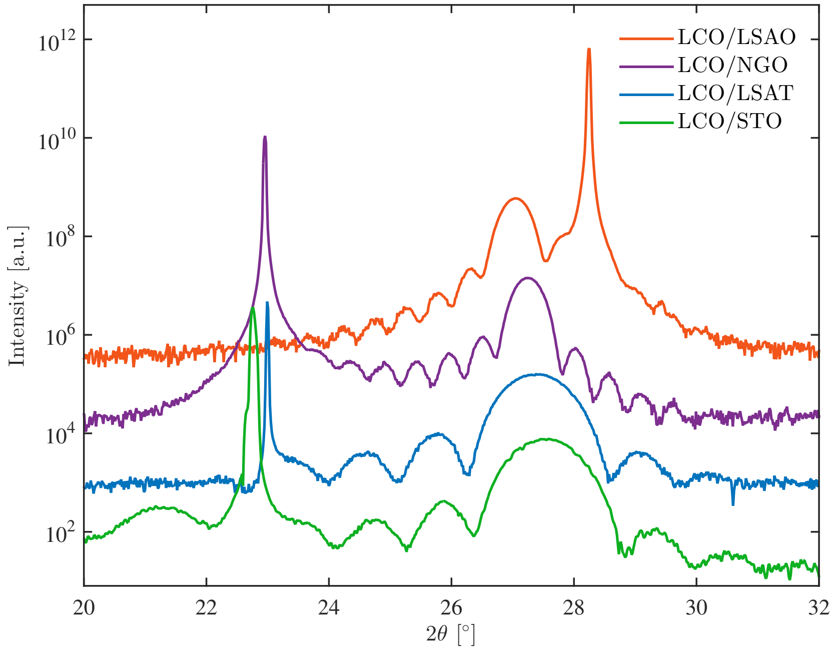

Supplementary Figure 3. **2θ scans of thin films.** X-ray diffraction measurements allowing the extraction of the c lattice parameter (main peak around $27° - 28°$) and the thickness for each sample as indicated. Sharp intense peaks around 23° and 28° belong to the substrates. Incident 1.5406 Å x rays were used for these measurements. Source data are provided as a Source Data file.

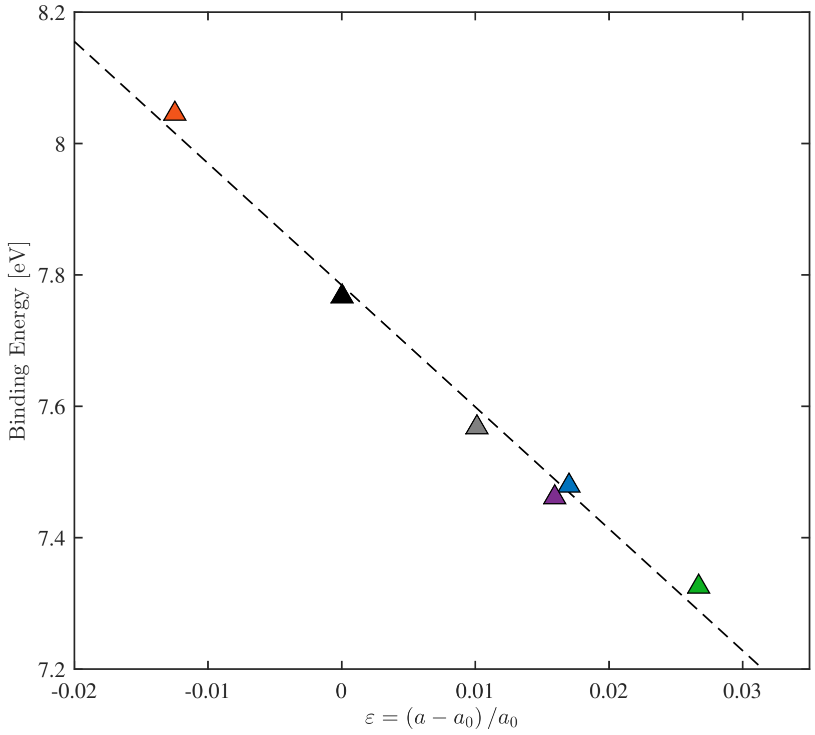

Supplementary Figure 4. **Evolution of the oxygen p bands versus strain.** Binding energies of the lower edge of the oxygen p bands (with p_x/p_y character), obtained from DFT calculations, as a function of strain ε. Source data are provided as a Source Data file.

II. SUPPLEMENTARY TABLE

Substrate	h [nm]	Doping [x]	a_S [Å]	a [Å]	c [Å]	T_c [K]	Ref.
$SrTiO_3$	15	0.10	3.905	3.80	13.17	10	[1]
$SrTiO_3$	50	0.15	3.905	$\sim$ 3.870	$\sim$ 13.16	$\sim$ 23.9	[2]
$SrTiO_3$	50	$\sim$ 0.16	3.905	—	$\sim$ 13.18	$\sim$ 23	[3]
$SrTiO_3$	200	$\sim$ 0.16	3.905	—	$\sim$ 13.20	$\sim$ 28	[3]
$SrTiO_3$	200	0.15	3.905	3.837	13.18	27.4	[2]
$NdGaO_3$	50	0.15	3.842	$\sim$ 3.797	$\sim$ 13.13	$\sim$ 18.2	[2]
$LaSrAlO_4$	15	0.10	3.754	3.76	13.31	49.1	[1]
$LaSrAlO_4$	50	0.15	3.756	$\sim$ 3.756	$\sim$ 13.26	$\sim$ 40.7	[2]
$LaSrAlO_4$	50	$\sim$ 0.15	3.756	—	13.29	$\sim$ 38	[3]
$LaSrAlO_4$	200	0.15	3.7564	3.762	13.29	43.8	[2]
Bulk							
—	—	0.10	—	3.778	13.21	27	[4]
—	—	0.15	—	3.777	13.23	36.5	[4]

Supplementary Table 1. **Structural thin-film parameters** *vs.* T_c. Lattice parameters (a and c) and superconducting transition temperature T_c for optimally doped $La_{2-x}Sr_xCuO_4$ thin films grown on substrates as indicated, as a function of thickness h. In-plane lattice parameters a_S of the respective substrates are indicated for completeness. Bulk $La_{2-x}Sr_xCuO_4$ is also presented for the dopings x considered for the thin films. All data are extracted from references as indicated in the last column. Source data are provided as a Source Data file.

[1] J. P. Locquet, J. Perret, J. Fompeyrine, E. Machler, J. W. Seo, and G. Van Tendeloo, "Doubling the critical temperature of $La_{1.9}Sr_{0.1}CuO_4$ using epitaxial strain," Nature **394**, 453–456 (1998).

[2] H. Sato and M. Naito, "Increase in the superconducting transition temperature by anisotropic strain effect in (001) $La_{1.85}Sr_{0.15}CuO_4$ thin films on $LaSrAlO_4$ substrates," Phys. C **274**, 221226 (1997).

[3] J. Locquet and E. Williams, "Epitaxially Induced Defects in Sr- and O-doped La_2CuO_4 Thin Films Grown by MBE: Implications for Transport Properties," Acta Phys. Pol. A **92**, 6984 (1997).

[4] H. Takagi, T. Ido, S. Ishibashi, M. Uota, S. Uchida, and Y. Tokura, "Superconductor-to-nonsuperconductor transition in $(La_{1-x}Sr_x)_2CuO_4$ as investigated by transport and magnetic measurements," Phys. Rev. B **40**, 2254–2261 (1989).
